# Detection of human cytomegalovirus in normal and neoplastic breast epithelium

**DOI:** 10.1186/2042-4280-1-8

**Published:** 2010-12-23

**Authors:** Lualhati E Harkins, Lisa A Matlaf, Liliana Soroceanu, Katrin Klemm, William J Britt, Wenquan Wang, Kirby I Bland, Charles S Cobbs

**Affiliations:** 1 Department of Pathology and Laboratory Medicine, Birmingham Veterans Hospital, 700 South 19th Street, Birmingham, AL, 35233, USA; 2 Research Institute, California Pacific Medical Center, (475 Brannan St., Suite 220) San Francisco CA, 94107, USA; 3 Pediatrics, University of Alabama at Birmingham, (1600 7th Avenue South), Birmingham AL, 35233 USA; 4 UAB Comprehensive Cancer Center Biostatistics Facility, University of Alabama at Birmingham, (1802 6th Avenue South), Birmingham AL, 35233 USA; 5 Surgery, University of Alabama at Birmingham, (1802 6th Avenue South), Birmingham AL, 35233 USA

## Abstract

**Introduction:**

Human cytomegalovirus (HCMV) establishes a persistent life-long infection, and can cause severe pathology in the fetus and the immunocompromised host[1]. Breast milk is the primary route of transmission in humans worldwide, and breast epithelium is thus a likely site of persistent infection and/or reactivation, though this phenomenon has not previously been demonstrated. Increasing evidence indicates HCMV infection can modulate signaling pathways associated with oncogenesis. We hypothesized that persistent HCMV infection occurs in normal adult breast epithelium and that persistent viral expression might be associated with normal and neoplastic ductal epithelium.

**Methods:**

Surgical biopsy specimens of normal breast (n = 38) breast carcinoma (n = 39) and paired normal breast from breast cancer patients (n = 21) were obtained. Specimens were evaluated by immunohistochemistry, in situ hybridization, PCR and DNA sequencing for evidence of HCMV antigens and nucleic acids.

**Results:**

We detected HCMV expression specifically in glandular epithelium in 17/27 (63%) of normal adult breast cases evaluated. In contrast, HCMV expression was evident in the neoplastic epithelium of 31/32 (97%) patients with ductal carcinoma in situ (DCIS) and infiltrating ductal carcinoma (IDC) cases evaluated (p = 0.0009).

**Conclusions:**

These findings are the first to demonstrate that persistent HCMV infection occurs in breast epithelium in a significant percentage of normal adult females. HCMV expression was also evident in neoplastic breast epithelium in a high percentage of normal and neoplastic breast tissues obtained from breast cancer patients, raising the possibility that viral infection may be involved in the neoplastic process.

## Introduction

Environmental and epidemiological factors that contribute to breast cancer are poorly understood, and only 5-20% of women with breast cancer are known to have hereditary risk factors [2]. While investigators have searched for viruses that contribute to breast cancer pathogenesis, no causal associations have been established [3]. An association of Epstein-Barr virus (EBV) with breast cancer has been reported in the literature [4-7]. These reports have principally relied upon use of the DNA detection techniques of PCR and Southern blot hybridization, but these data have not been validated with appropriately sensitive in situ techniques. Other investigators have found that human endogenous retroviruses with homology to mammary tumor virus are associated with a significant percentage of breast cancer, and can contribute to epithelial cell transformation both *in vitro *and *in vivo*[8-13]. Human papilloma virus (HPV) is a known oncogenic virus that has been detected in breast cancer cell lines and breast tumor cells[14-17]. The viral proteins E6 and E7 are capable of immortalizing normal human breast epithelial cells, however it is unclear whether HPV has a causal role in breast cancer[18].

Increasing evidence in the last 10 years suggests that human cytomegalovirus (HCMV) is associated with several human malignancies, including malignant glioma, colorectal carcinoma, prostate cancer, and skin cancer, and that HCMV gene products can modulate oncogenic properties of cells in vitro [19-26]. HCMV gene products can dysregulate cell cycle progression, cause DNA mutations, block apoptotic pathways, inhibit immune response, and inhibit tumor suppressor protein functions [27-33]. Thus, cells that are persistently or abortively infected with HCMV might be at increased risk of developing genomic instability and immunological privilege, which could accelerate neoplastic transformation.

While evidence of HCMV in human breast glandular tissues is lacking, breast glandular epithelium is a likely reservoir for persistent HCMV infection in humans. Breast milk represents an established primary route of HCMV transmission in humans, and the shedding of cell free virus occurs in the breast milk of over 90% of women who are seropositive for HCMV [34-38]. Since persistent HCMV infection of breast epithelium could, in theory, promote malignant transformation of infected breast epithelium, we sought to determine the detection of HCMV gene products in normal and neoplastic breast. To this end, we used highly sensitive immunohistochemical (IHC) and in situ hybridization techniques (ISH) to analyze archived paraffin-embedded non-neoplastic breast tissues (from reduction mammoplasty patients) and breast carcinoma specimens with matched non-neoplastic appearing breast tissues for evidence of HCMV antigens and nucleic acids. This report is the first to demonstrate that persistent HCMV infection occurs in a significant percentage of non-neoplastic breast tissues. Moreover, we find that HCMV infects a very high percentage of both non-neoplastic and neoplastic breast epithelium from patients with breast cancer.

## Materials and methods

### Clinical Samples

Formalin-fixed, paraffin-embedded, surgical biopsy specimens were obtained from histopathologically normal breast tissues (from patients undergoing elective breast reduction mammoplasty with no known history of breast cancer), neoplastic and non-neoplastic adjacent breast tissues from breast cancer patients from the pathology tissue procurement archives of the University of Alabama at Birmingham and the Birmingham Veterans Affairs Hospital. The ages of breast cancer patients ranged from 22-80, (mean = 48). Ages ranged from 20-57 in the normal breast (reduction mammoplasty) group (mean = 36). All samples were obtained in accordance with ethics guidelines from the institutional review board of each institution. A faculty pathologist (K. K.) re-examined all cases to confirm histological diagnosis. Since these paraffin embedded specimens were part of the patient record, we were not allowed to exhaust the specimen. Some specimens had only limited amounts of tissue available, and we were therefore not able to perform all of the immunohistochemical and in situ hybridization studies on each specimen. For this reason the total number of specimens used exceeded the number analyzed for any given reagent.

### Immunohistochemical analyses of paraffin sections

4-μm paraffin sections were procured from biopsy specimens of neoplastic and non-neoplastic breast, deparaffinized in xylene, and hydrated them in graded alcohols. Samples were processed as previously described [25] using monoclonal antibodies for immunodetection (anti-IE1/2 ["IE"; IgG_1 _isotype, 1:40, Chemicon, Temecula, CA], anti-CMV cocktail, containing antibodies specific for early and late antigens ["E/L"; IgG_2α _isotype,1:40, Innovex Biosciences, Richmond, CA], and anti-CMV late antigen ["L"; IgG_2α _isotype, 1:40, Chemicon, Temecula, CA]). As controls we used anti-CD34 (1:40, BioGenex), and anti-smooth muscle actin (1:40; BioGenex, San Ramon, CA), and omission of primary antibody (no antibody). Immunostaining with the different antibodies was performed in a blinded fashion with respect to tissue diagnosis. One pathologist (K. K.) who was blinded to the antibody used analyzed immunostaining results and sections were called positive if specific immunoreactivity was detected.

### In situ hybridization of paraffin sections

To confirm that HCMV nucleic acids were present in the pathological sections, the investigators performed in situ hybridization with a commercially available HCMV oligonucleotide cocktail probe labeled with fluorescein (BioGenex/Innogenex, San Ramon, CA). This probe consisted of six fluorescein isothiocyanate (FITC)-conjugated 40-mer probes spanning coding regions within the HCMV IE1 gene, and did not hybridize with human DNA sequences. Positive control probe specific for human Alu DNA sequences and negative control probe specific to an insect virus genome, both provided by the manufacturer, were also used. 4-μm paraffin sections were cut, deparaffinized, and hydrated through an established series of graded ethanol. Status of fixation was assessed for all cases before proceeding, and sections were post-fixed in formalin if necessary. After treatment, the prepared slides were rinsed in distilled water, dehydrated to 100% ethanol and air dried. Prediluted probe was then placed onto the sections, a cover slip was applied and slides were denatured on a MISHA thermocycler (Shandon Lipshaw/Hybaid Omnigene) at 90°C for 8-10 minutes, and then hybridized at 37°C in humidified chamber overnight. Slides were washed in TBST buffer, subjected to probe wash (0.05% SSC buffer 20 min. at 40°C), then washed in 1× PBS. Endogenous avidin, biotin and Fc receptors were then blocked using avidin/biotin blocking kit (BioGenex, San Ramon, CA) and Fc block (Innovex Biosciences, Richmond, CA). Fluorescein-labeled probe was then detected using Supersensitive^® ^in situ detection system (Innogenex, San Ramon, CA) with the chromogen BCIP/NBT.

### PCR and DNA sequencing

DNA was purified from paraffin sections (3-6 10 μm sections) cut from a subset of the same biopsy specimens described above using DNeasy Tissue System (Qiagen, Valencia, CA) per manufacturer's instructions. To avoid potential PCR contamination, these experiments were performed in a laboratory devoid of previous exposure to infectious or recombinant HCMV. All preparations were processed throughout in a blinded fashion; no positive controls were used in any PCR reactions and blank paraffin blocks were cut sequentially between each patient sample and processed identically. For preparation of each case, the sectioning blade was replaced and the cutting surface was cleansed with xylene and ethanol. From each sample, 100-250 ng of DNA was amplified by nested PCR using internal and external primers specific for HCMV glycoprotein B (UL55) gene as described [39]. Samples were considered positive when a band of 140 bp size could be visualized on agarose gel with ethidium bromide. Amplified DNA products were visualized on 1.5% agarose gels with ethidium bromide, bands were cut out, and DNA was extracted (gel extraction kit, Qiagen, Valencia, CA) and analyzed by automated sequencing (ABI Model 377 DNA Sequencer, Foster City, CA). Confirmation of HCMV sequence was performed using a NCBI Blast search.

### Statistical analyses

Immunohistochemical data were determined in breast tumor samples, breast tumor control samples, and normal control samples. The percentages of positive sections for each specific monoclonal antibody in each group of samples were estimated. Chi-square test or Fisher's exact test was applied to compare tumor samples or tumor controls samples to normal control samples.

## Results

### Immunohistochemistry for HCMV

To determine whether HCMV was present in breast epithelium from normal controls and breast cancer patients, archived formalin fixed paraffin embedded breast tissues were utilized. Non-neoplastic breast tissues from breast reduction mammoplasty patients was used as "non-neoplastic normal" control (N = 38). To evaluate HCMV presence in neoplastic tissues, the investigators obtained tumor biopsy specimens (N = 39) and histopathologically non-neoplastic biopsy specimens from many of the same patients ("tumor control", N = 21).

**Figure 1:**
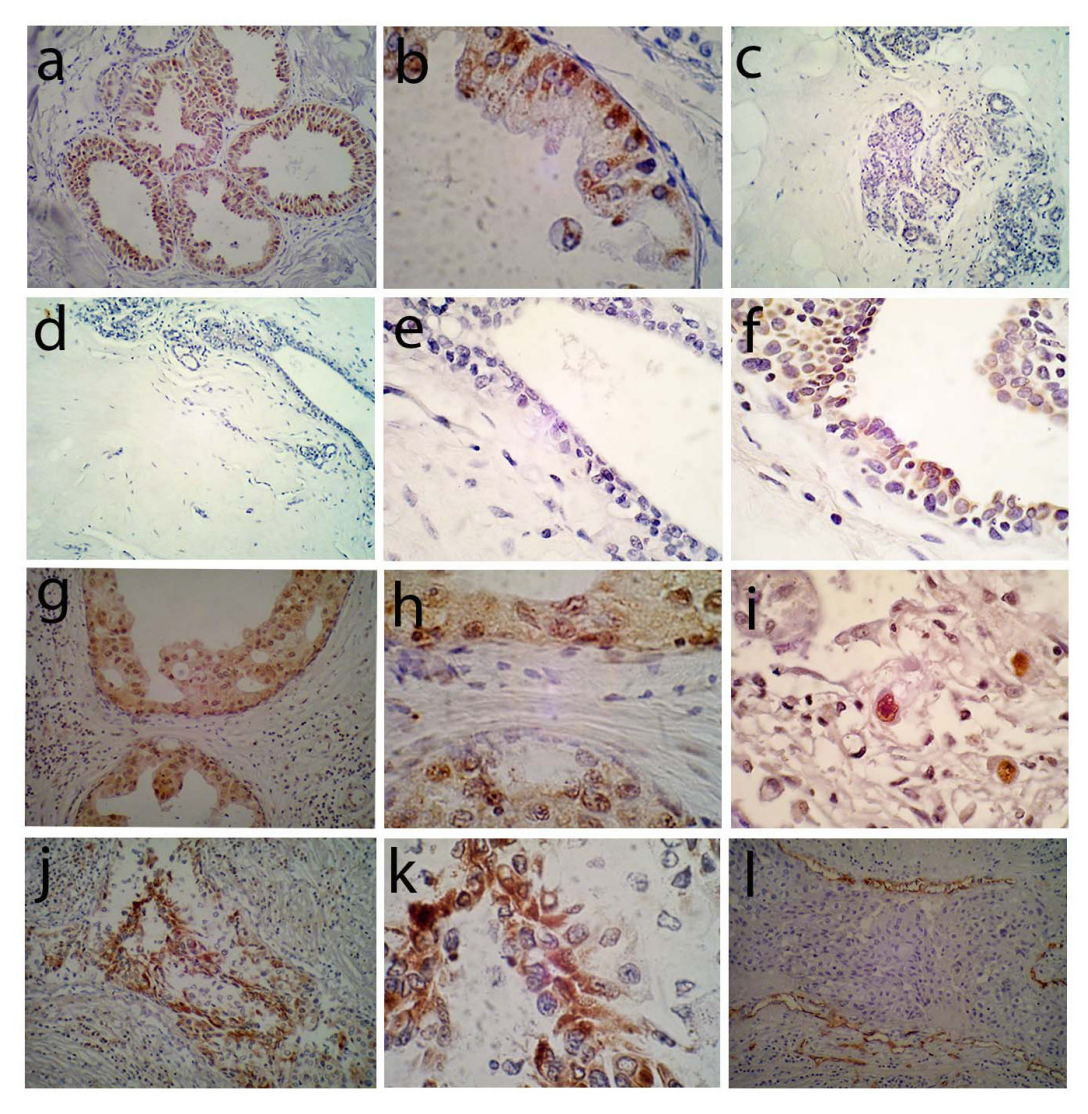
**Immunohistochemical detection of HCMV in normal breast and breast cancer**. (a, b) Examples of HCMV IE immuonreactivity in isolated area of normal ductal epithelium in normal breast from a reduction mammoplasty patient are presented. Low (a, 40×) and higher (b, 100×) power views of the same area of immunoreactive epithelium demonstrate discrete perinuclear and cytoplasmic epithelial cell staining. No IE immunoreactivity is detected in two different specimens obtained from normal reduction mammoplasty (c and d, 40×; e is 100× magnification of d). IE immunoreactivity is demonstrated primarily in a nuclear distribution in matched non-neoplastic epithelium from a patient with infiltrative ductal carcinoma (f, 100×). Early and late (E/L) immunoreactivity is demonstrated in the tumor epithelial cells, but not the stroma, from an area of ductal carcinoma in situ (DCIS) in a patient with infiltrative ductal carcinoma (g, 40×, h, 100×). Positive control immunostaining for IE immunoreactivity is shown in HCMV infected pneumocytes from an AIDS patient with CMV pneumonia (i, 100×). Late antigen (L) immunoreactivity is shown in another patient with infiltrative ductal carcinoma (j, 40×; k, 100×). Negative control (IE isotype control antibody staining for CD34) immunoreactivity is seen only in vascular endothelial cells in an area of infiltrative ductal carcinoma (l, 40×).

**Figure 2:**
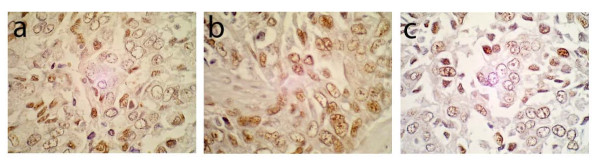
**Immunohistochemical detection of three different HCMV antigens in a serial sections from infiltrative ductal carcinoma**. Immunoreactivity with monoclonal antibodies specific for HCMV immediate early (IE; a), early and late (E/L; b), and late (L; c) antigens is shown in serial sections from a single specimen of infiltrative ductal carcinoma (100×).

To detect HCMV protein expression, we performed immunohistochemistry on as many specimens as possible with a panel of monoclonal antibodies that were specific to HCMV antigens expressed at different stages of the viral life cycle. Due to limitations on the use of archived patient specimens and/or tissue quality, not all specimens could be evaluated for all antibodies and in situ hybridization. The antibodies used are specific for HCMV immediate early ("IE"), early and late ("E/L"), or late antigens ("L") (Figures 1 and 2). As controls, we used monoclonal antibodies specific for smooth muscle actin (SMA) (data not shown) and CD34 (Figure 1, l). These antibodies react to smooth muscle cells and vascular endothelial cells, respectively, but not breast epithelial cells, and serve as IgG_2α _and IgG_1 _isotype positive and negative monoclonal antibody controls, respectively. As additional negative controls, we performed immunostaining in the absence of primary antibody for all cases (data not shown).

We detected HCMV-IE antigens in neoplastic epithelium from 97% of breast cancers tested while IE antigens were detected in only 63% (p = 0.0009) of normal control cases tested (Table 1, Figure 1). IE immunoreactivity occurred in normal appearing ductal epithelium (Figure 1, a-b), epithelium in DCIS, and in IDC epithelial cells (Figure 2, a) evident in tumor specimens, but IE immunoreactivity was not observed in stromal cells (Figure 1, a-f). Immunoreactivity with the HCMV-E/L monoclonal antibody cocktail was detected in 84% of breast cancer specimens tested; only 21% of normal breast controls had confirmed immunoreactivity (p < 0.0001; Table 1). The HCMV-E/L immunoreactivity was detected in a similar cellular pattern as that of IE antigen, with a staining pattern restricted to epithelium (Figure 1, g-h; Figure 2, b). Monoclonal antibody immunoreactivity with HCMV-L was detected in 56% of breast cancer specimens and 39% of normal controls (p = 0.227; Table 1). The pattern of late antigen cellular localization was also similar to IE and E/L immunoreactivity (Figure 1, j, k; Figure 2, c). HCMV late antigen was detected, in general, at a less intense level, although occasional rare cells were very intensely positive for late antigen (Figure 2, c). In some of the tumors there were areas of homogenous low level immunoreactivity to IE, E/L and L antigens, while in other tumors there were scattered foci of positive tumor cells. The intensity of immunostaining varied significantly from tumor cell to tumor cell within any given tumor (e.g., Figure 1, j, k; Figure 2).

**Table 1:** Comparisons of HCMV immunoreactivity in epithelium between breast cancer and non-neoplastic breast tissue from breast reduction mammoplasty.

	Tumor (N = 39)			Normal Control (N = 38)			p of Chi-Square test
	N (examined)	+		N (examined)	+		
		n	%		n	%	
IE	32	31	97	27	17	63	0.0009
E/L	25	21	84	28	6	21	<0.0001
L	27	15	56	28	11	39	0.2270
No Ab	19	0	0	20	0	0	--
CD34	16	0	0	9	0	0	--
Actin	28	0	0	29	0	0	--

Specific immunoreactivity to HCMV immediate early (IE) antigen and HCMV early and late (E/L) antigens were significantly higher in breast cancer cases than normal controls. Immunoreactivity to the HCMV late (L) antigen was higher in breast cancer than normal breast. No immunoreactivity was detected in any specimen when primary antibody was omitted. All specimens had specific immunoreactivity to vascular endothelial cells and smooth muscle cells with anti-CD34 and anti-smooth muscle actin antibodies, respectively, but no immunostaining of epithelial cells was observed with these antibodies.

In general, we detected little HCMV immunohistochemical staining of stromal fibroblasts in the tumor cases. We did, however, perform immunostaining on a subset of tumor cases with a monoclonal antibody specific to the HCMV pp65 tegument antigen. In several cases we detected intense immunostaining of infiltrating stromal macrophages with this antibody (see Additional file 1).

Immunoreactivity was not detected in breast epithelium when primary antibody or monoclonal antibodies specific for smooth muscle actin and/or CD34 were used (Table 1), although immunoreactivity was evident in smooth muscle cells and endothelial cells with these antibodies as expected (e.g., Figure 1, l). The HCMV-IE, E/L and L monoclonal antibodies were specific for HCMV antigens when tested against known HCMV infected lung tissues from an AIDS patient (e.g, Figure 1, i).

Our analysis also demonstrated that there was a higher incidence of HCMV immunoreactivity in "tumor control" tissues from breast cancer patients than in "normal control" tissues from breast reduction mammoplasty (Table 2). Interestingly, while the incidence of HCMV IE immunoreactivity trended to a higher level in the breast cancer patients' control tissues compared to the normal control tissues, it was not statistically significant. The incidence of HCMV E/L and L immunoreactivity was, however, significantly greater in the tumor control breast tissue compared to the normal control tissues (Table 2).

**Table 2:** Comparisons of HCMV immunoreactivity between nonneoplatic breast tissue from known cancer patients and non-neoplastic breast tissue from breast reduction mammoplasty normal conrols.

	Tumor Control (N = 21)			Normal Control (N = 38)			p of Chi-Square test
	N (examined)	+		N (examined)	+		
		n	%		n	%	
IE	13	12	92	27	17	63	0.0678*
E/L	15	11	73	28	6	21	0.0009
L	13	10	77	28	11	39	0.0249
No Ab	9	0	0	20	0	0	--
CD34	6	0	0	9	0	0	--
Actin	8	0	0	29	0	0	--

* Two-sided Fisher's exact test

Specific immunoreactivity to HCMV E/L antigens was significantly higher in nonneoplastic breast from breast cancer cases than from breast reduction mammoplasty controls. Immunoreactivity to the HCMV late (L) antigen was also significantly higher in breast cancer than normal breast.

Of the tumor specimens and matched breast control specimens from tumor patients, a total of 30 specimens were analyzed with all three antibodies (25 tumor cases and 5 matched controls). 22 of the true normal breast tissues (from reduction mammoplasty) were also analyzed with all three antibodies. In all but one case where all three antibodies were tested in the tumor specimens, the specimens were positive for IE antibody staining (Table 1). In 3 of these cases both the E/L and L antibody staining was negative, otherwise it was present. In 2 other cases either E/L or L antibody staining was negative while the other two antibodies were found to be positive.

In contrast, while 15 of the 22 true normal breast specimens from reduction mammoplasty patients were positive for IE antibody staining, only 7 of these 15 cases were also immunoreactive for E/L and L. These findings suggest that HCMV early and late antigen expression is less frequently found in normal breast tissues than in tumor tissues or normal appearing breast tissues from breast cancer patients.

To determine if the HCMV positivity rate of the tissue samples was due to the differences in the mean age or ethnicity of the control versus the cancer patient populations, we analyzed the data by assessing the HCMV IE1 immuonreactivity rate of patients ≤45 years or >45 years of age. In ≤45 years group, 7 of 8 (87.5%) cancer patients had positive HCMV IE1 antigen, compared to 12 positives of 17 (70.6%) normal tissue, and the difference was not significant. In >45 years group, all 20 cancer patients were positive for HCMV IE1 antigen, compared to 4 positives out of 7 normal tissues (p = 0.012). Thus, these data remained significant for patients >45 years of age.

There were 7 African-American patients and 31 Caucasian patients in the breast cancer group. There were 11 African-American and 21 Caucasian patients in the normal breast reduction mammoplasty group. For Caucasians, there was no significant difference between HCMV prevalence (95.7% in cancer tissues compared to 88.9% in normal tissues). For African-Americans, HCMV prevalence was 100% in cancer tissues, while it was 50% in the normal tissues. However, this difference was not significant because of small numbers.

### In situ hybridization

We performed in situ hybridization on breast cancer specimens and their paired control breast specimens, as well as normal controls from individuals with no history of breast cancer, to determine the presence of HCMV nucleic acids. We detected HCMV nucleic acids specifically in neoplastic or nonneoplastic epithelial cells in 16/18 randomly selected patients from the breast cancer pool. In 3/3 cases that we tested both neoplastic and control epithelium from the same patient, HCMV nucleic acids were detected in both specimens. We also performed in situ hybridization on 18 randomly selected "normal control" breast specimens, and 11/18 of these specimens confirmed specific HCMV nucleic acid detection.

**Figure 3:**
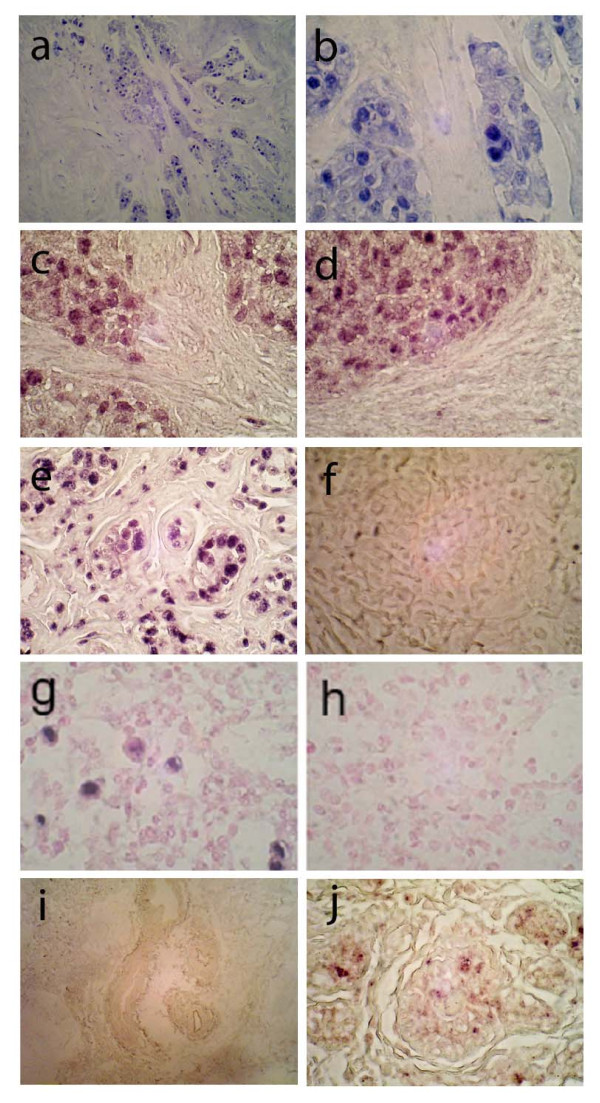
**HCMV in situ hybridization in infiltrating ductal carcinoma**. Two examples of HCMV ISH staining from two different patients with infiltrating ductal carcinoma are displayed. Low and high power images of the first patient (a, 40×; b, 100×) demonstrate heterogeneous pattern of detection of HCMV nucleic acids in the nuclei (dark blue) and cytoplasm (light blue) in infiltrating tumor cells but no significant nucleic acid detection in the intervening stroma. Two images from another example of infiltrating ductal carcinoma (c, d; 100×) also reveal areas of HCMV nucleic acid detection in nuclear (dark purple) and cytoplasmic (light purple) areas, without any significant nucleic acid detection in the intervening stroma. Positive control probe specific for DNA Alu repeats from the same case reveals intense (dark purple) nuclear probe hybridization with nucleic acids in infiltrating tumor nuclei and intervening stromal cells, without evidence of cytoplasmic staining (e, 100×). Hybridization signal of a negative control probe specific to insect nucleic acids is completely absent in tumor tissues (f, 100×). Positive control (HCMV infected lung) is positive for HCMV nucleic acid hybridization in scattered pneumocytes (blue cells in g, 100×), while negative control probe is not detected in the same specimens (h, 100×; light hematoxylin counterstain was used in g and h). Low power image (i, 40×) of normal breast epithelium (from reduction mammoplasty) that was negative for HCMV nucleic acid hybridization reveals no hybridization signal. High power (j, 100×) image of normal breast epithelium from reduction mammoplasty reveals faint purple hybridization signal in scattered normal ductal epithelial cells.

The pattern of HCMV immunoreactivity was similar to the pattern of ISH staining observed. We detected HCMV nucleic acid hybridization in normal breast epithelium and in neoplastic epithelium in areas of DCIS and IDC, but HCMV nucleic acids were principally undetectable in stromal cells (Figure 3, a-d). The pattern of HCMV nucleic acid detection within the cell was distinctly different from that of the Alu positive control probe, which is entirely specific to cellular nuclear DNA Alu repeats (Figure 3, e). In HCMV positive cells, we detected HCMV nucleic acids in the nucleus but also predominantly in the cell cytoplasm (as shown in Figure 3, b-d, and see Additional file 2). A positive control DNA probe (specific to DNA Alu repeat sequences) was specific for nuclear DNA in both epithelial and stromal cells in DCIS/IDC in tumors and normal breast (Figure 3, e), while a non-specific DNA probe was non-reactive in tumor and normal tissues (Figure 3, f). The specificity of HCMV nucleic acid hybridization was confirmed by detection of HCMV infected cells in HCMV infected lung tissues from an AIDS patient (Figure 3, g), while a negative control probe was negative in these same tissues (Figure 3, h). In normal breast epithelium from reduction mammoplasty patients, the amount of signal from HCMV nucleic acid hybridization was, in general, dramatically less than that found in breast tumor cells (e.g., Figure 3, i-j)

With respect to the internal consistency of immunohistochemical staining for HCMV antigens and detection of HCMV nucleic acids by in situ hybridization, there was a high correlation between positive for IHC and ISH specimens among the breast cancer and paired controls from breast cancer patients. 16/18 cases analyzed were positive for both HCMV antigen and nucleic acids, while in 2 cases there was a discordance (see Additional file 3).

In the normal breast tissue from non-cancer controls, there was a high degree of inconsistency between IHC and ISH results. Some of the positive IHC cases were negative for ISH, and vice versa (see Additional file 3). We attribute these results to the overall extremely low levels of antigen and nucleic acids detected in these normal tissues compared to the cancer cases. At such low levels of antigen and nucleic acid detection, we suspect that limitations of detection may have resulted in lack of internally consistent results between the two groups. In addition, it is possible that latent HCMV infection may occur in normal breast epithelium, in which case nucleic acids may be detected in the absence of protein expression.

### PCR and Sequencing

We performed nested PCR for HCMV UL55 gene using DNA that was extracted from paraffin sections of 8 tumors and 4 control cases. 6/8 tumor cases and 1/4 normal control cases demonstrated amplified HCMV UL55 gene, which was confirmed by direct sequencing of the PCR products (not shown). Since the tumor specimens that were tested were all positive for HCMV by immunohistochemistry, we hypothesize that the negative PCR specimens may have had viral nucleic acids below the level of detection for our assay, or viral genetic material may have been more degraded than cellular housekeeping genes used as controls, since the DNA quality in these assays is very variable. Alternatively, the paraffin specimens used for these assays, which were not necessarily sequential sections to the ones used for immunohistochemistry and in situ hybridization, may have had less viral genome present. The one case of normal breast control tissue that was positive for HCMV UL55 by PCR was also positive for HCMV by immunohistochemistry and in situ hybridization, while the remaining three cases were negative by all three detection methods.

## Discussion

Breastfeeding is the major route of HCMV transmission during the first year of life in countries where most women are seropositive and breast-feed their infants [36]. Since cell free virus is shed in the breast milk in virtually all HCMV seropositive females, the natural reservoir for HCMV in the breast is likely breast glandular epithelial cells. We demonstrate here that 17/27 of the normal breast specimens in our study from females with no history of breast cancer exhibited evidence of persistent HCMV infection as determined by HCMV-IE antigen expression.

Unexpectedly, we found that 31/32 (97%) of cases of breast carcinoma in our study also have evidence of HCMV infection and expression based upon immunohistochemistry. Immunoreactivity to non-IE HCMV antigens was detected in a significantly higher percentage of breast cancer specimens than normal breast cases. Overall, these data indicate that persistent HCMV infection occurs specifically in breast glandular epithelium for a significant percentage of normal adult females and that HCMV IE protein expression is significantly associated with neoplastic compared to nonneoplastic breast glandular epithelium in patients over age 45 in our group.

Our data are consistent with a previous PCR-based report that indirectly suggested HCMV infection is present in breast cancer [40]. In this study, the investigators analyzed 12 specimens of normal breast from a non-cancer group, and 62 samples of invasive ductal carcinoma from breast cancer patients for several DNA viruses using DNA PCR followed by Southern hybridization [40]. The viruses analyzed included human papillomavirus (HPV), HCMV, EBV, herpes simplex virus 1 (HSV-1), HSV-2, and human herpesvirus - 8 (HHV-8). Of these six DNA viruses, only HCMV was detected in normal breast (8/12; 67%) specimens. HCMV DNA was also detected in 47/62 (76%) of invasive ductal carcinoma specimens. Since in situ techniques were not used in this study, no clear conclusion that HCMV was located in tumor epithelial cells could be made.

Our novel findings of the expression of the IE antigen and other gene products in normal and neoplastic breast epithelial cells indicate that the breast epithelium is a reservoir for persistent HCMV infection. While this phenomenon has not been previously demonstrated, it is not completely unexpected. HCMV is known to be able to infect multiple organs, including the salivary glands, lung, gastrointestinal tract, kidney, liver, spleen and brain [41-43]. The best candidate cells for latent infection are thought to be monocytes [44]. However, chronic infection and expression of HCMV gene products in normal breast epithelium may represent a critical component of the viral life-cycle, since breast milk is a major mode of transmission and survival for the virus.

A well known consequence of persistent viral infection and inflammation is neoplastic transformation. Indeed, an increasing percentage of human malignancies in the last several decades have been attributed to chronic infection and chronic inflammation [45]. It is well established that chronic inflammation plays a critical role in the transition from neoplastic precursor to full-blown invasive malignancy, and inflammation is considered the seventh hallmark of neoplasia [46-48]. This period of chronic inflammation may indeed be essential for the neoplastic process in malignancy, and may be facilitated by infectious agents that act as "promoters". For example, Hepatitis C virus chronically infects the liver and causes a persistent inflammatory immune response resulting in hepatoma[49]. Another example is Epstein Barr virus (EBV) in nasopharyngeal carcinoma. EBV is ubiquitous in the human population, and thus is not oncogenic under normal circumstances. However, EBV infection in the nasopharynx of individuals exposed to certain environmental carcinogens is critical in the development of nasopharyngeal carcinoma, through expression of latent EBV genes that promote cell growth and survival [50].

An accumulating body of evidence indicates that HCMV gene expression in normal epithelial cells, tumor cells and tumor infiltrating macrophages could promote an oncogenic environment. Multiple HCMV gene products are known to promote mutagenesis and to dysregulate cell cycle checkpoint controls, and drive oncogenic signaling pathways (reviewed in [26]). Recent experimental evidence has shown that the chronic expression of TNF-alpha and IL-1 beta in the pre-malignant microenvironment in the setting of inflammation can produce dramatic increases in the likelihood of malignant transformation via activation of the NF-kB transcriptional activator [51]. Furthermore, two critical downstream effectors of this NF-kB pathway with respect to oncogenicity appear to be COX-2 and IL-6 [49,52,53]. IL-6 induction and expression in tumor cells and tumor associated myeloid cells has an important role in chronic inflammatory oncogenic signaling, likely by activation of the STAT-3 transcriptional activator [54]. Hence, the NF-kB pathway has a dual effect in tumor promotion by preventing cell death of cells with malignant potential and by stimulating pro-inflammatory cytokines in infiltrating myeloid and lymphoid cells. Chronic HCMV infection could potentially promote these important oncogenic signaling pathways since HCMV infection expresses a chemokine receptor US28, which has oncogenic potential and has been shown to signal through the NF-kB pathway and activate downstream COX-2, STAT-3 and IL-6 expression [55,56]. Indeed, an etiological role for HCMV in breast cancer has been hypothesized based on epidemiological considerations, and investigators have demonstrated that breast cancer patients have increased IgG antibody titers to HCMV compared to controls [57,58].

## Conclusion

The data presented here indicate that HCMV infection occurs in normal breast epithelium in a majority of adult females evaluated and that a high percentage of breast cancer specimens have evidence of HCMV infection. These findings suggest that further research in this area is warranted to determine whether HCMV infection of breast epithelium represents an important factor in the initiation and promotion of breast cancer, and raise the possibility that in the future, antiviral based strategies may play a role in the management of this disease.

## List of Abbreviations

DCIS: ductal carcinoma in situ; EBV: Epstein Barr Virus; IDC: infiltrating ductal carcinoma; IE: Immediate early; IHC: immunohistochemistry; ISH: in situ hybridization; HCMV: human cytomegalovirus; L, late; IE/L, immediate early and late.

## Competing interests

The authors declare that they have no competing interests.

## Authors' contributions

LH performed the immunohistochemistry and in situ hybridization studies. LM assisted in writing and preparing data for the study. KK scored slides for immunoreactivity. WW participated in the design of the study and performed the statistical analysis. WB, LS and KB helped in the study design and drafting of the manuscript. CC conceived of the study, and participated in its design and coordination, analyzed the data and drafted the manuscript. All authors have read and approved the final manuscript.

## Supplementary Material

Additional file 1 **Immunohistochemical detection of pp65 in infiltrating ductal carcinoma**. Low and high power views of an infiltrating ductal carcinoma demonstrating immunoreactivity to the HCMV pp65 tegument antigen (pp65 mAb; Novocastra). Low power (a, 20×) demonstrates immunoreactivity (brown staining) in tumor cells but not intervening stroma. However, intense immunoreactivity of macrophages was to pp65 was detected in some cases in the outer stromal layer (scattered brown cells). Higher power view (b, 40×) demonstrates diffuse nuclear and cytoplasmic pp65 immunoreactivity in infiltrating ductal tumor cells. Click here for file

Additional file 2 **HCMV in situ hybridization of normal breast epithelium in infiltrative ductal carcinoma**. HCMV nucleic acid detection in an area of matched normal breast epithelium from a patient with infiltrative ductal carcinoma reveals nuclear and cytoplasmic HCMV nucleic acid detection in epithelial cells but not in adjacent stromal cells (a, 100×). In an adjacent section, positive control hybridization to Alu DNA repeats reveals intense nuclear signal from ductal epithelium, basement membrane and stromal cells, but not cytoplasmic staining (b, 100×). Click here for file

Additional file 3 **Comparison of HCMV detection methods from breast cancer samples and matched controls**. Correlation of results of immunostaining and in situ hybridization for breast cancer cases and matched controls (A), and normal breast tissues from patients with reduction mammoplasty (B) are reported. Click here for file
