# Supplementary figures and images for: Detection of human cytomegalovirus in normal and neoplastic breast epithelium
Source: Herpesviridae. 2010 Dec 23;1:8. doi: 10.1186/2042-4280-1-8 (PMC3063230; doi:10.1186/2042-4280-1-8)

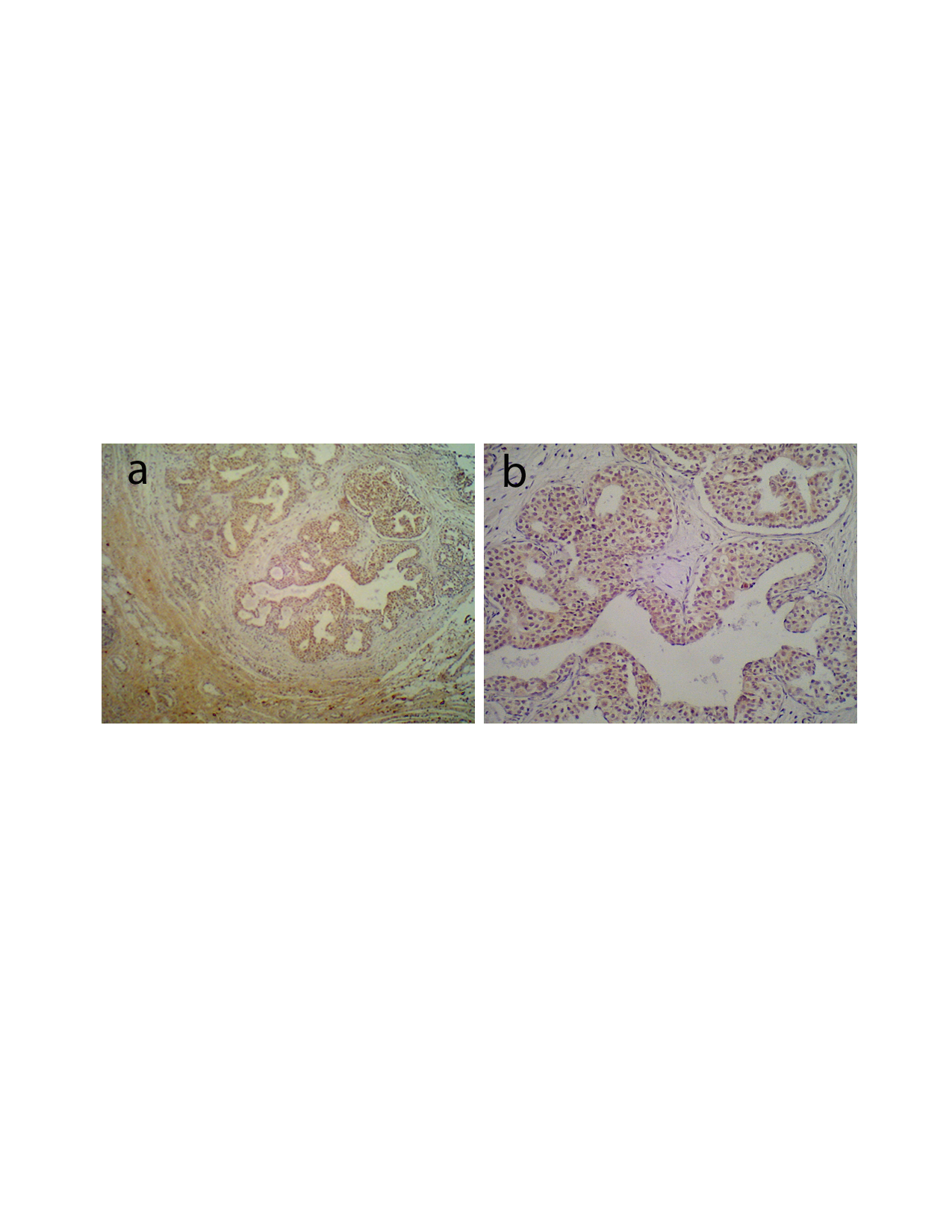

Supplement: Additional file 1 — Immunohistochemical detection of pp65 in infiltrating ductal carcinoma. Low and high power views of an infiltrating ductal carcinoma demonstrating immunoreactivity to the HCMV pp65 tegument antigen (pp65 mAb; Novocastra). Low power (a, 20×) demonstrates immunoreactivity (brown staining) in tumor cells but not intervening stroma. However, intense immunoreactivity of macrophages was to pp65 was detected in some cases in the outer stromal layer (scattered brown cells). Higher power view (b, 40×) demonstrates diffuse nuclear and cytoplasmic pp65 immunoreactivity in infiltrating ductal tumor cells. [file 2042-4280-1-8-S1.TIFF]

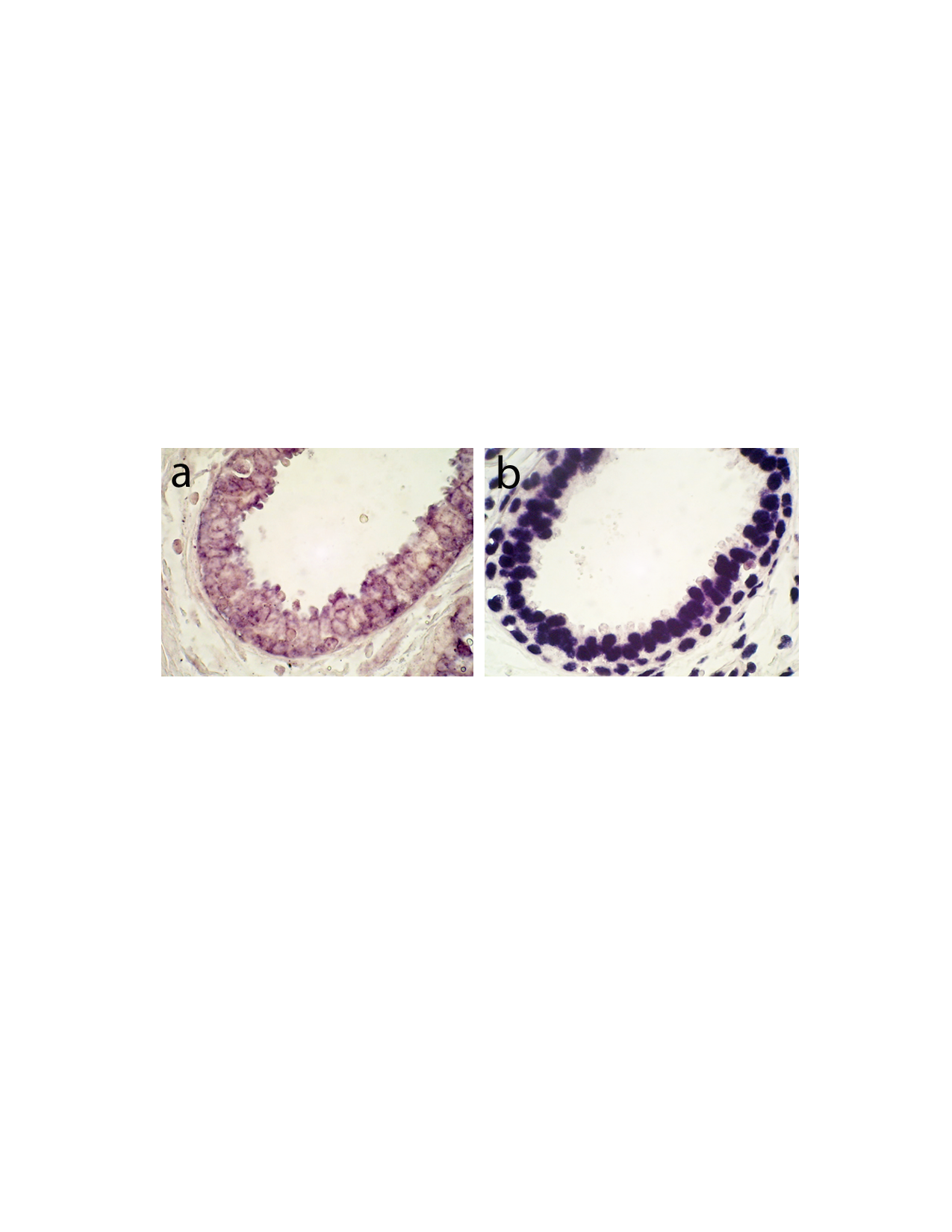

Supplement: Additional file 2 — HCMV in situ hybridization of normal breast epithelium in infiltrative ductal carcinoma. HCMV nucleic acid detection in an area of matched normal breast epithelium from a patient with infiltrative ductal carcinoma reveals nuclear and cytoplasmic HCMV nucleic acid detection in epithelial cells but not in adjacent stromal cells (a, 100×). In an adjacent section, positive control hybridization to Alu DNA repeats reveals intense nuclear signal from ductal epithelium, basement membrane and stromal cells, but not cytoplasmic staining (b, 100×). [file 2042-4280-1-8-S2.TIFF]
