# Supplementary material for: Detection of human cytomegalovirus in normal and neoplastic breast epithelium
Source: Herpesviridae. 2010 Dec 23;1:8. doi: 10.1186/2042-4280-1-8 (PMC3063230; doi:10.1186/2042-4280-1-8)
Supplement: Additional file 3 — Comparison of HCMV detection methods from breast cancer samples and matched controls. Correlation of results of immunostaining and in situ hybridization for breast cancer cases and matched controls (A), and normal breast tissues from patients with reduction mammoplasty (B) are reported. [file 2042-4280-1-8-S3.DOC]

A. Breast cancer cases and paired non-neoplastic breast

Case # Dx IHC ISH

**1. IDC + +**

**1. NL + +**

2. IDC + -

3. IDC + +

4. IDC + +

5. DCIS + +

**6. IDC - +**

**6. NL + +**

7. IDC + +

8. IDC + +

9. IDC + +

10. IDC + +

11. IDC + +

12. IDC + +

13. IDC + +

14. IDC + +

15. IDC + +

16. IDC + +

IDC = infiltrative ductal carcinoma; NL = matched non-neoplastic breast from same patient

B. Normal breast tissue from patients with no history of breast cancer

Case # Dx IHC ISH

1. nnl - +

2. nnl + -

3. nnl + -

4. nnl + -

5. nnl + +

6. nnl + +

7. nnl - -

8. nnl - -

9. nnl - +

10. nnl + +

11. nnl - +

12. nnl + -

13. nnl - +

14. nnl - +

15. nnl + -

16. nnl + +

17. nnl + +

18. nnl + +

nnl = non-neoplastic normal breast epithelium
